# The Natural History and Clinical Outcomes of Transmembrane Protein 43 Cardiomyopathy: A Systematic Review

**DOI:** 10.3390/jcm14165611

**Published:** 2025-08-08

**Authors:** Annagrazia Cecere, Marika Martini, Maria Bueno Marinas, Ilaria Rigato, Alessandro Parodi, Kalliopi Pilichou, Barbara Bauce

**Affiliations:** Department of Cardiac, Thoracic, and Vascular Sciences and Public Health, University of Padova, 35128 Padova, Italy; annagrazia.cecere@unipd.it (A.C.); marika.martini.1@phd.unipd.it (M.M.); maria.buenomarinas@unipd.it (M.B.M.); ilaria.rigato@unipd.it (I.R.); alessandro.parodi90@gmail.com (A.P.); kalliopi.pilichou@unipd.it (K.P.)

**Keywords:** transmembrane protein 43 (*TMEM43*), arrhythmogenic cardiomyopathy, ventricular arrhythmias, sudden cardiac death

## Abstract

**Background:** Arrhythmogenic cardiomyopathy (ACM) is an inherited heart disorder characterized by structural and functional myocardial alterations, often accompanied by ventricular arrhythmias (VAs), which may ultimately result in sudden cardiac death (SCD). While mutations in genes coding for desmosomal components are commonly identified in affected individuals, genetic variants involving non-desmosomal proteins have recently been recognized as contributors to the disease’s etiology. In 2008, a mutation in the transmembrane protein 43 (*TMEM43*) was identified as being responsible for a fully penetrant, sex-related, and severe form of ACM. This review aimed to systematically synthesize the current evidence on the natural history, electrocardiographic, and imaging findings as well as the clinical outcomes of *TMEM43* cardiomyopathy. **Methods:** A systematic search was performed in the PubMed, Scopus, and Web of Science databases, following the PRISMA guidelines, using the terms “TMEM43” AND “cardiomyopathy”. After an initial screening of 144 retrieved articles, 80 were considered relevant. Upon a full-text review and eligibility assessment, 12 studies involving 903 individuals harboring TMEM43 variants were selected for inclusion. **Results:** Male patients more frequently carried the pathogenic *TMEM43* variant (*n* = 505, 55.9%) and exhibited an earlier arrhythmic onset of the disease (33.2 years old versus 46.2 years old in female patients), supporting the need for earlier implantable cardioverter–defibrillator implantation (30.4 versus 42.2 years old). Palpitations, chest pain, and syncope were the most common presenting symptoms. Baseline electrocardiograms commonly demonstrated poor R wave progression, QRS prolongation, and premature ventricular contractions (PVCs). Arrhythmic events, including malignant VAs and SCD, were early manifestations of the disease, especially in male patients. Frequent PVCs and left ventricular dilation were considered early markers of the disease and were predictive of arrhythmic events. Conversely, heart failure was reported as a late clinical outcome, requiring heart transplantation in a minority of cases (1.5%). **Conclusions:** *TMEM43* cardiomyopathy is a fully penetrant autosomal dominant form of ACM, characterized by a well-defined clinical phenotype that is more severe and presents earlier in male patients.

## 1. Introduction

Arrhythmogenic cardiomyopathy (ACM) is an inherited myocardial disease, clinically characterized by ventricular morpho-functional abnormalities and electrical instability [1]. The first clinical manifestation of the disease frequently includes ventricular arrhythmias (VAs) and, in some cases, sudden cardiac death (SCD), most commonly emerging during the second or third decade of life. Given the variability in its phenotypic expression, a multiparametric evaluation is recommended to establish a diagnosis [1].

According to the most recent Padua Classification and the latest European Guidelines on Cardiomyopathies, genetic testing has become increasingly important to both the diagnostic process and the prognosis of ACM patients [2,3]. Detecting a pathogenic or likely pathogenic (P/LP) variant in a gene associated with ACM is considered a major diagnostic criterion. This finding supports a diagnosis in individuals with borderline clinical features or predominant left ventricular (LV) involvement and facilitates the early detection of mutation carriers within affected families [3].

Mutations in desmosomal proteins, which are responsible for mechanical interactions between myocytes, have been shown to be involved in approximately 50% of ACM index cases [4]. Beyond desmosomal proteins, several non-desmosomal components involved in the complex myocyte interaction network have been linked to ACM [4]. However, their pathogenic role remains uncertain, highlighting the need for focused studies to clarify their contribution to the disease.

**Figure 1:**
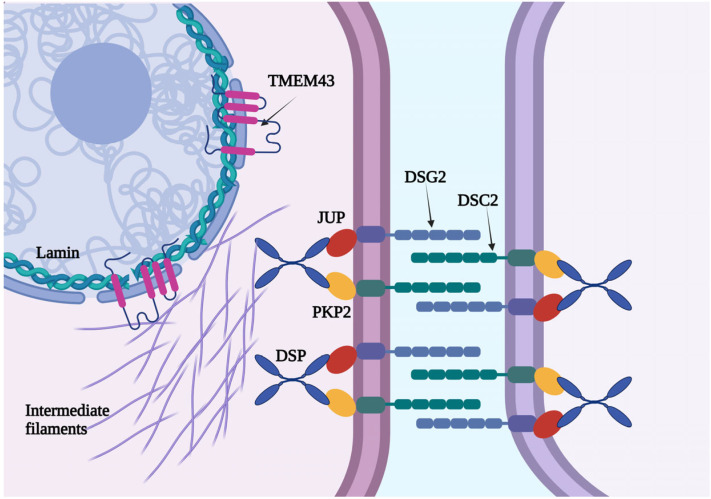
A schematic representation of TMEM43, a nuclear protein with four transmembrane domains and a large hydrophilic domain.

Transmembrane protein 43 (TMEM43) is a protein with four transmembrane domains and a large hydrophilic domain. Although its function has not been fully clarified, TMEM43 is a nuclear protein that interacts with lamin and emerin and has also been localized in the endoplasmic reticulum (Figure 1).

The missense variant p.S358L has been shown to impair the intercalated disc and gap junction integrity across different model systems [5]. Located on chromosome 3p25 (MIM 604400), TMEM43 has been designated as the causal gene for ARVD5. The missense mutation TMEM43-p.S358L has been identified as a founder mutation responsible for a severe, sex-related, and fully penetrant ACM phenotype in the Newfoundland population [6].

Through a systematic approach, this review sought to assess the natural history and electrocardiographic and imaging findings as well as the clinical outcomes of TMEM43 cardiomyopathy.

## 2. Method

### 2.1. Protocol Disclosure and Identification

This systematic review was prospectively registered in PROSPERO (CRD420251001758), the international database maintained by the Centre for Reviews and Dissemination, the University of York (York, UK).

### 2.2. The Search Strategy and Study Selection

This systematic review was conducted in accordance with the PRISMA guidelines (http://www.prismastatement.org/, accessed on 25 February 2025). A comprehensive search of the PubMed, Scopus, and Web of Science databases was performed to identify clinical studies investigating TMEM43-associated cardiomyopathy. The review was guided by specific clinically focused areas, including TMEM43; cardiomyopathy; ventricular arrhythmias; ventricular dysfunction; sudden cardiac death; electrocardiographic and imaging findings; and implantable cardioverters–defibrillators. The search strategy used the terms “TMEM43” AND “cardiomyopathy”, adapted to the appropriate MeSH terms and free-text keywords for each database. The reference lists of all included articles were manually screened to capture additional studies relevant to the review objectives. Furthermore, the “Related Articles” feature on PubMed was explored to identify potentially eligible literature. No restrictions on the publication date or study design were applied to ensure a comprehensive selection of the relevant literature. Titles and abstracts of articles published in English were evaluated, and the full texts of potentially eligible studies were reviewed in detail to extract original data and examine reference citations. The literature search was updated to 25 February 2025.

### 2.3. Study Eligibility Criteria

According to the aim of this systematic review, only original research articles written in English were included. Eligible studies were required to involve human subjects carrying TMEM43 mutations or to provide clinical data relevant to TMEM43-related cardiomyopathy. So, experimental studies conducted in vitro or in animal models were considered only when they offered translational insights with potential clinical relevance.

Articles were excluded if they were review papers, editorials, conference abstracts, or single case reports. Studies that did not primarily focus on TMEM43 or that lacked direct implications for phenotype, natural history, or clinical outcomes were also excluded from the final selection.

### 2.4. Extraction of the Study Data

**Figure 2:**
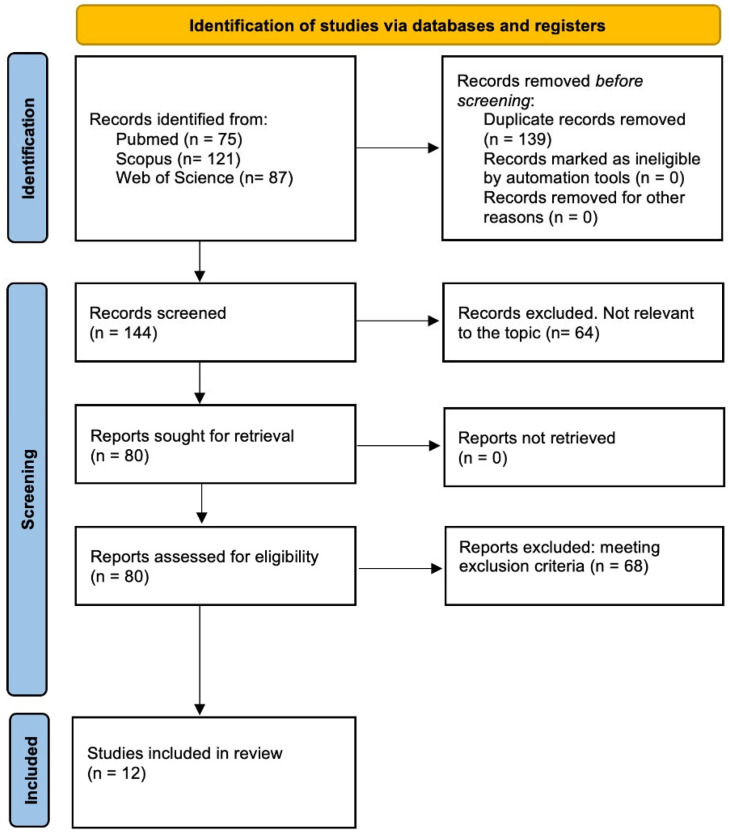
PRISMA flow diagram illustrating study identification, screening, and selection process.

Two reviewers (A.C. and B.B.) independently extracted data from the selected studies. Any discrepancies were resolved through a discussion within the review team. A detailed overview of the search strategy and the study selection process is illustrated in Figure 2. The extracted information included the article title, first author’s name, year of publication, and country of origin of the study population and a qualitative description of the target cohort. Each eligible study underwent a thorough evaluation to retrieve all relevant data and confirm the eligibility of individual patients.

In line with the objectives of this systematic review, the following data were collected: the presence of the genetic variant and its prevalence within the ACM cohort, the onset of disease, sex differences in phenotypical expression, clinical manifestations, electrocardiograms (ECGs), 24 h Holter monitoring and electrophysiologic study (EPS) findings, and echocardiographic and cardiac magnetic resonance (CMR) characteristics. Clinical outcomes, in terms of death, SCD, heart failure (HF), heart transplantation (HF), and implantable cardioverter–defibrillator (ICD) discharge, were also considered. The statistical analysis was conducted using version 29.0.2.0 of SPSS (SPSS Inc., Chicago, IL, USA).

## 3. Results

The main results of the studies included in this systematic review are reported in Table 1.

### 3.1. Study Retrieval

A total of 283 titles were retrieved (75 from PubMed, 121 from Scopus, and 87 from Web of Science). After duplicate removal, 144 records were screened, resulting in the identification of 80 potentially relevant studies. A total of 68 studies were excluded after a full-text assessment due to non-fulfillment of the inclusion criteria. Ultimately, twelve papers were included in this systematic review (Table 1) [4,6,7,8,9,10,11,12,13,14,15,16].

A PRISMA flow diagram was used to summarize the literature research and selection process for this review (Figure 2).

### 3.2. The Clinical Phenotype of TMEM43-Associated Cardiomyopathy

A total of 12 studies comprising 903 patients carrying a pathogenic TMEM43 variant were included in the analysis [4,6,7,8,9,10,11,12,13,14,15,16]. The mean age at presentation was 40.5 years. Males were more frequently affected and carriers of a TMEM43 variant (*n* = 505, 55.9%), showing an earlier onset of the disease (33.2 years versus 46.2 years in female patients). Clinical data were available in 180 patients [8,14]. Palpitations were the most common early clinical manifestation (*n* = 136, 75.6%), followed by chest pain (*n* = 71, 39.4%) and syncope (*n* = 63, 35%).

An ICD was implanted in 296 patients (33%), with 245 of them (82.8%) receiving it for primary prevention. The age at ICD implantation was lower in males than that in females (30.4 versus 42.2 years).

### 3.3. Electrocardiographic Characteristics of TMEM43-Related Cardiomyopathy

A total of nine studies reported baseline ECG findings, including data from 522 patients (57.8%) [4,6,8,9,12,13,14]. Poor R wave progression (PRWP), defined as an R wave < 3 mm in lead V3, was evaluated in two studies and identified in 75 patients (14.4%) [8,13]. A low QRS voltage was reported in both limb leads (*n* = 7, 1.3%) and precordial leads (*n* = 4, 0.8%). Premature ventricular contractions (PVCs) in baseline ECGs were described in 47 patients (9%) [8]. QRS prolongation (>110 msec) was considered in two studies and reported in 12.6% of patients [6,8].

Twenty-four-hour Holter monitoring was performed in seven studies, including a total of 433 patients [4,6,7,8,9,13,14]. The presence and severity of VAs were also analyzed. Specifically, 198 patients (45.7%) had ≥200 PVCs per 24 h, and 161 patients (37.2%) had ≥1000 PVCs per 24 h. Non-sustained ventricular tachycardia (NSVT) was reported in 93 patients (21.5%).

Signal-averaged ECGs (SAECGs) were analyzed in three studies. In a total of 199 pts, 83 (41.7%) presented positive SAECGs [6,7,13].

An EPS was performed in 11 patients across two studies, revealing biventricular and endo-epicardial low-voltage areas in 9 (81.8%) and 4 pts (36.4%), respectively [11,16].

### 3.4. The Imaging Features of Patients with TMEM43 Cardiomyopathy

Imaging features were assessed using both echocardiography (*n* = 454 patients) [4,6,7,8,9,12,13,14] and CMR (*n* = 59 patients) [4,7,9,14,15,16]. LV dysfunction was observed in 47 patients (10.3%), with a mean echocardiographic left ventricular ejection fraction (LVEF) of 50.7%. Accordingly, LV dilation was reported in 201 patients (44.3%). Conversely, right ventricular (RV) involvement, assessed as dysfunction and dilation, was reported in 29 (6.4%) and 26 (5.7%) patients, respectively. Biventricular involvement was identified in 23 patients (5.1%).

CMR was used to assess the presence of myocardial fibrosis through the identification of late gadolinium enhancement (LGE) and was analyzed in six studies [4,7,9,14,15,16]. LGE was reported in 20 patients (34%), showing a subepicardial (*n* = 12, 60%), midmural (*n* = 2, 10%), and ring-like pattern (*n* = 5, 25%). Additionally, LGE was observed in the RV in seven patients (35%).

### 3.5. Clinical Outcomes in Patients with TMEM43 Cardiomyopathy

Outcome data were reported in ten studies, including a total of 891 patients (99%) [6,7,8,9,10,11,12,13,14,15]. Death occurred in 180 patients (20.2%), with SCD accounting for 131 cases (14.7%). HF was observed in 27 patients (3%), and among them, 13 (48%) required a HT. Eighty-nine patients experienced an ICD discharge (10%). One study reported the composite clinical outcomes for patients with mutations in a nuclear membrane gene [16].

## 4. Discussion

ACM is a rare genetic disease, typically inherited as an autosomal dominant transmission, although rare autosomal recessive forms have also been described [17]. The natural history of the disease is characterized by a marked arrhythmic phenotype, including malignant VAs and SCD, along with progressive deterioration of biventricular systolic function. The mortality rate varies between 0.08 and 3.6%/year in different series [1]. The phenotype seems to be more aggressive in men, both in terms of an early onset of clinical manifestations and a greater severity of symptoms.

ACM was initially described as a disease with almost exclusive involvement of the RV; however, advances in pathological characterization, genetic insights, and the broader use of CMR have shown that LV involvement is possible—either predominantly, as in left-dominant forms, or concurrently with the RV in biventricular phenotypes [2].

Desmosomal proteins, including *plakoglobin* (*JUP*), *desmoplakin* (*DSP*), *plakofillin* (*PKP2*), *desmoglein 2* (*DSG2*), and *desmocollin* (*DSC2*), play a crucial role in homeostasis of the cardiac myocytes and have been extensively implicated in the pathogenesis of the disease [4]. These proteins are responsible for cell-to-cell adhesion and myocyte attachment. Therefore, impairment of desmosomal protein function can destabilize the intercellular junctions, triggering myocyte detachment and death [4]. Secondly, desmosomal proteins mediate intracellular and intercellular signal transduction.

Several in vitro studies have demonstrated that abnormal plakoglobin protein may induce the activation of nuclear adipogenic and fibrotic gene pathways, contributing to myocardial fibro-fatty scar formation [18].

In recent years, recent evidence has implicated several non-desmosomal proteins—including TMEM43, Desmin (DES), Phospholamban (PLN), Filamin C (FLNC), Cadherin 2 (CDH2), and Tight Junction Protein 1 (TJP1)—in the pathogenesis of ACM [4].

The *TMEM43* gene encodes *LUMA*, a nuclear-envelope-associated transmembrane protein. *LUMA* closely interacts with nuclear envelope proteins, including lamin and emerin [19], and is therefore involved in connecting between the nuclear lamina and the cytoskeleton [9]. Merner et al. were the first to identify a missense mutation, *TMEM43-p.S358L*, in a Newfoundland cohort. This mutation is responsible for a fully penetrant, autosomal dominant transmitted disease characterized by markedly severe, sex-related clinical manifestations [6].

### 4.1. The Clinical Characteristics of TMEM43-Associated Cardiomyopathy

The review of the 12 selected studies highlights consistent epidemiological and clinical features in TMEM43 mutation carriers, aligning with the original description of the disease phenotype produced by Merner et al. [6]. Sex and age at disease onset are crucial factors in understanding the natural history of the disease. In particular, ten studies assessed the age of disease onset in male and female patients, confirming an early onset of clinical manifestations in the male cohort [4,6,7,8,9,10,11,12,13,14]. Although symptoms were available only in 180 patients, the most frequently reported clinical manifestations were palpitations, chest pain, and syncope [8,14].

Approximately one quarter of the enrolled patients underwent an ICD implantation, as reported in five studies [7,9,10,12,13,14]. In three of these studies, ICD implantation was considered an inclusion criterion, with primary prevention of SCD being the most frequent indication [7,10,13]. Hodgkinson et al. demonstrated a substantial benefit of ICD implantation in male mutation carriers, whereas this benefit appeared to be less evident in females [7,10].

Given the full penetrance of the disease, the severe arrhythmic phenotype, and its demonstrated clinical benefit in reducing SCD, ICD implantation has been suggested for male mutation carriers by the age of 18 years [8,10,13]. Conversely, in female mutation carriers, ICD implantation may be considered in the presence of cardiac abnormalities, particularly frequent PVCs [6,7,8]. Accordingly, the age at ICD implantation was found to be lower in males compared to that in females.

### 4.2. Electrocardiographic Features and VAs in Patients with TMEM43 Cardiomyopathy

**Figure 3:**
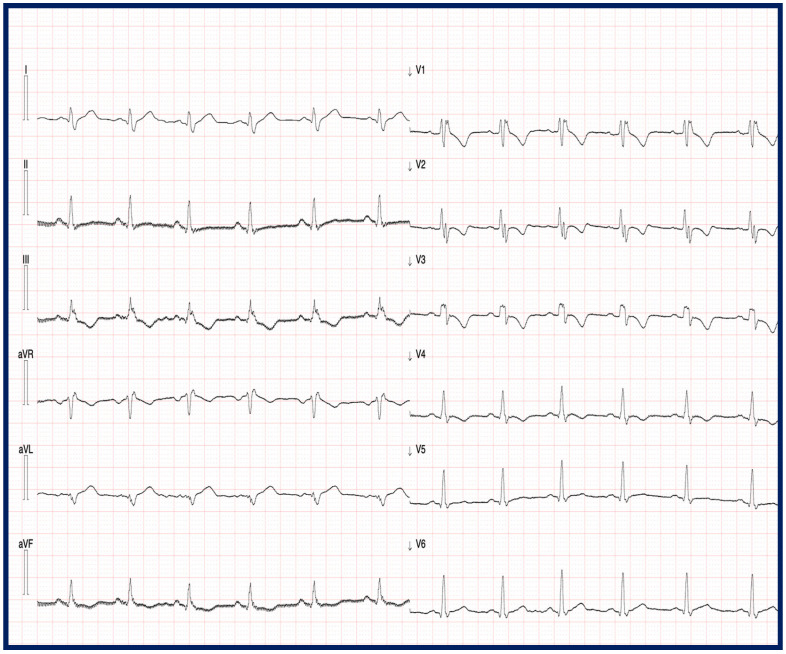
Electrocardiographic features of a 40-year-old man with TMEM43 mutations, symptomatic for exertional dyspnea. The ECG shows a sinus rhythm with a right branch bundle block. Negative T waves are present in D3, aVF, and V1–V4.

Electrocardiographic data were available in 57.8% of the evaluated patients [4,6,8,9,12,13,14,15,16]. Patients with TMEM43 cardiomyopathy exhibited distinct electrocardiographic characteristics, including PRWP, QRS prolongation, and the presence of PVCs on baseline ECGs. In contrast to desmosomal variants of ACM, low limb lead voltages were reported in only a minority of patients [14,16]. Cabrera-Borrego et al. further reported that patients with TMEM43 mutations more frequently presented with a left bundle branch block (LBBB) than a right bundle branch block (RBBB) (Figure 3) [16].

Accordingly, the morphology of ventricular tachycardia (VT) in pts with TMEME43 mutations more frequently exhibits an LBBB morphology [16]. Similarly to the classical phenotype of ACM associated with desmosomal protein mutations (*PKP2* and *DSG2*), an arrhythmic substrate in the RV has also been found in patients with *TMEM43* mutations [16]. It has been hypothesized that *TMEM43* could impact *DSG2* solubility, partially explaining the similar clinical phenotype of these two variants, both characterized by a high incidence of severe arrhythmic events [20].

The analysis of 24 h Holter monitoring revealed that patients with *TMEM43* mutations presented frequent (in the same studies, PVCs ≥ 1000/24 h) and complex ventricular ectopy [4,6,7,8,9,13,14]. In particular, Hodgkinson et al. demonstrated that the presence of ≥200 PVCs in 24 h Holter monitoring was the earliest arrhythmic manifestation in both sexes, with an earlier onset in male patients (25 years versus 48 years in female patients) [8]. Accordingly, in males, a ventricular ectopic burden ≥1000 PVCs/24 h was the only independent clinical predictor of ICD therapy; no such predictors were observed in the female cohort [10].

SAECGs were performed in a minority of patients [6,7,13] and were revealed to be abnormal in almost half of patients.

EPSs were carried out in only two studies [11,16], with both confirming the marked arrhythmic phenotype of the disease that requires an interventional approach. In particular, AbdelWahab et al. evaluated the outcomes of catheter ablation in patients with and without *TMEM43* mutations, demonstrating a more severe form of the disease in mutation carriers, characterized by biventricular involvement and increased inducibility of VT [11]. VT recurrence was also reported in *TMEM43* carriers, suggesting progression of the disease rather than an incomplete procedure [21].

### 4.3. The Imaging Findings in Patients with TMEM43 Cardiomyopathy

Imaging findings, obtained using echocardiography, CMR, or both, were analyzed in all of the studies included in this systematic review. LV dilation appeared as an early sign of the disease, followed by a reduction in LVEF reduction [6]. Biventricular involvement was a frequent finding. One particularly interesting study investigated the echocardiographic longitudinal myocardial deformation in a family with a *TMEM43* mutation [14]. These researchers found that the global longitudinal strain, along with the LVEF, was more impaired in genotype-positive, phenotype-positive patients compared to genotype-positive, phenotype-negative individuals [14].

Due to its independence from the patient’s acoustic window and its ability to provide multiple reconstruction planes, CMR provides a more accurate morpho-functional evaluation of the biventricular dimensions and function [22]. In *TMEM43* patients, CMR has proven particularly useful in characterizing abnormalities in regional contractility, especially in the RV [15,16].

Matos et al. reported that *TMEM43* cardiomyopathy is a progressive condition, and CMR was able to detect the progression of LV dysfunction during follow-up [15]. Consistent with the updated diagnostic criteria for ACM, CMR is pivotal in detecting ventricular myocardial fibrosis, the key anatomical substrate for arrhythmogenesis [2,3].

Myocardial fibrosis, as detected using CMR through LGE, presents high concordance with endomyocardial biopsy findings and enables the identification of different clinical variants of ACM, including right-dominant, left-dominant, and biventricular phenotypes [2,3]. According to the latest 2023 ESC Guidelines on Cardiomyopathy, CMR should be considered in genotype-positive relatives with normal cardiac function but an elevated SCD risk in order to improve myocardial tissue characterization [3].

**Figure 4:**
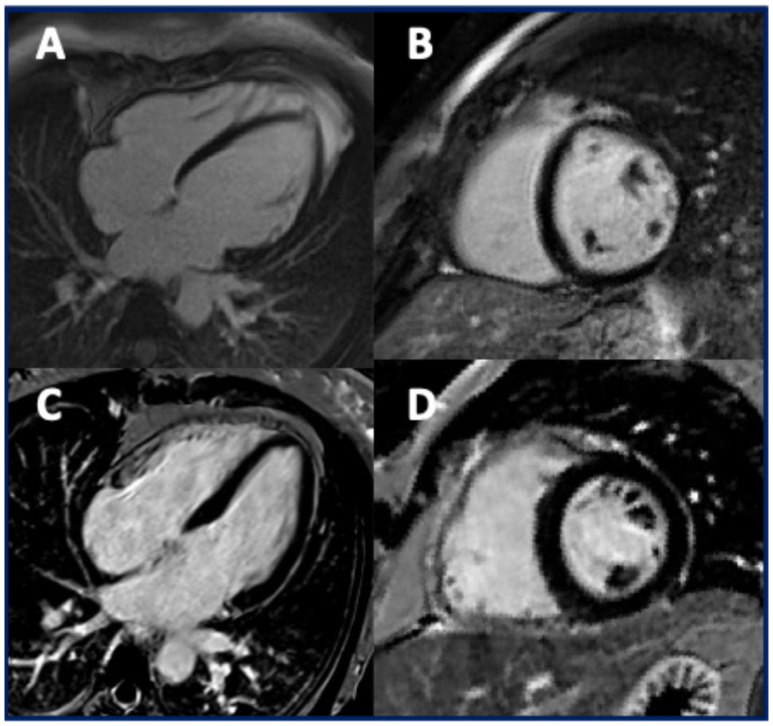
CMR images of two patients carrying TMEM43 mutations. In the superior panel (**A**,**B**), the CMR findings in a 40-year-old man with TMEM43 mutations, symptomatic for exertional dyspnea, are shown. A dilated left ventricle with mildly reduced systolic function is present, without evidence of biventricular fibrosis (**A**,**B**: post-contrast 4-chamber long-axis and short-axis images). In the inferior panel (**C**,**D**), CMR images of an asymptomatic 42-year-old female carrying a TMEM43 mutation are shown. The patient exhibits normal biventricular dimensions and function, with no signs of myocardial fibrosis (**C**,**D**: post-contrast 4-chamber long-axis and short-axis images).

In TMEM43 cardiomyopathy, patients may exhibit a non-ischemic pattern of LGE, most commonly in the subepicardial region; however, myocardial fibrosis may be absent in some cases (Figure 4) [15,16].

A ring-like LGE pattern has also been reported, although less frequently than in cardiomyopathies associated with *DSP* or *FLNC* mutations [23]. Biventricular LGE was commonly observed [15]. However, considering that the arrhythmic substrate during EPS was predominantly located in the RV, Cabrera-Borrego et al. raised concerns about the accuracy of CMR in guiding EPS procedures [16]. Further studies are needed to characterize myocardial fibrosis in patients with *TMEM43* mutations better, including the use of advanced post-processing software dedicated to scar identification.

### 4.4. The Clinical Outcomes in Patients with TMEM43 Cardiomyopathy

*TMEM43* cardiomyopathy is characterized by a well-defined clinical and instrumental phenotype. First, male patients consistently exhibited worse clinical outcomes, both in terms of disease severity and earlier onset [8]. Secondly, PRWP, PVCs, and LV dilation were frequently observed as markers of the disease [6,8,13].

The main outcomes associated with *TMEM43* cardiomyopathy are arrhythmic events, which typically occur at an early stage, and HF. ICD discharges, regarded as instances of aborted SCD, were reported in five studies [7,10,13,14,15]. Paulin et al. identified male sex, the presence of >1000 PVCs on 24 h Holter monitoring, and LV dilation as strong predictors of SCD in TMEM43 patients strongly associated with arrhythmic risk [13]. Accordingly, the main predictors of SCD in TMEM43 mutation carriers include (1) male sex and (2) in females the presence of one or more of the following risk factors: LV systolic dysfunction (LVEF < 45%), NSVT, myocardial fibrosis on CMR, or >200 PVCs on 24 h Holter monitoring [8,10].

HF is typically a late clinical manifestation of TMEM43 cardiomyopathy, requiring HT in a minority of cases [8]. Hodgkinson et al. reported that male survivors often developed a dilated cardiomyopathy phenotype, which may be attributed to the interaction of *TMEM43* with nuclear envelope proteins such as lamin and emerin [8]. Mutations in the *LMNA* gene are well-established causes of dilated cardiomyopathy: this interaction may also help explain the development of this phenotype in *TMEM43* patients [24].

Due to the small number of *TMEM43* mutation cases (*n* = 6), Cabrera-Borrego et al. analyzed the clinical outcomes in patients with nuclear gene mutations (*TMEM43* and *LMNA*), revealing a high recurrence of VT and HF, compared with those with desmosomal or cytoskeleton gene mutations [16].

Interestingly, two studies have evaluated the impact of physical exercise on patients with *TMEM43* mutations [12,13]. Dominguez et al. investigated this effect in three Spanish families, only revealing a trend toward increased VAs in women [12]. This finding suggests that exercise may act as a disease modifier that is potentially more evident in females, in whom the natural progression of the disease is generally slower. In contrast, Paulin et al. conducted a study assessing the impact of physical exercise on *TMEM43* patients who have received a primary prevention ICD implantation [13]. They demonstrated that high-level physical activity (>9.0 MET-hours/day) in the year prior to ICD implantation was significantly associated with an increased risk of malignant VAs. The detrimental role of physical exercise in *TMEM43* patients may be explained by increased stiffness of the cell nucleus, promoted by an abnormal connection with the cytoskeleton, which makes the ventricles more vulnerable to the wall stress induced by exercise [9,20]. For these reasons, the 2023 ESC Guidelines do not recommend high-intensity exercise, including competitive sports, in individuals carrying *TMEM43* mutations (Class of recommendation III, level of evidence C) [3].

#### Limitations

*TMEM43* is responsible for a rare form of ACM [1,2]. Two studies included in this systematic review focused on both desmosomal and non-desmosomal genes associated with different ACM genocopies [4,16]. In particular, a composite clinical outcome was reported for patients with nuclear gene mutations (*TMEM43* and *LMNA*) [16]. Additionally, one study considered both pathogenic variants and variants of unknown significance of *TMEM43* [15]. Electrocardiographic and imaging findings were reported in the majority of the studies, although the variables assessed were often heterogeneous (for instance, the presence/absence of LV dilation or quantification of the LV’s dimensions).

## 5. Conclusions

TMEM43 cardiomyopathy is a rare form of ACM characterized by a fully penetrant autosomal dominant inheritance and sex-related differences in phenotypic expression, including severe arrhythmic events and LV dysfunction.

Further studies are needed to understand the complex interaction between *TMEM43* and cardiac myocytes better in order to enable a genotype-specific approach to diagnosis and risk stratification in affected patients.

## Figures and Tables

**Table 1:** Overview of included studies.

Citation	Aims	Patients with TMEM43 Mutation (NM_024334.3)	HGVSc	HGVSp	Conclusions
**Hodgkinson KA, JACC 2005 [7]**	To evaluate the impact of ICD implantation in ARVD5 patients with 3p25-linked autosomal dominant disease vs controls	48 pts with ARVD5 linked to 3p25 (obligate carriers, *n* = 8; DNA haplotype, *n* = 36; SCD or VT, *n* = 4)	NA	NA	ICDs significantly reduced the mortality in male patients with an ARVD5 linked to 3p25.
**Merner ND, Am J Hum Genet 2008 [6]**	To identify the ARVD5 gene and conduct a penetrance analysis of clinical features	257 affected pts (144 mutation carriers and 113 clinically affected patients)	c.1073C>T	p.Ser458Leu	Mutation at locus ARVD5 causes a lethal, fully penetrant, sex-influenced cardiomyopathy.
**Hodgkinson KA, Clin Genet 2013 [8]**	To determine the phenotype and natural history of TMEM43-related ARVC	258 affected pts (genetic mutation, *n* = 83; obligate carrier, *n* = 118; SCD or VT, *n* = 57)	c.1073C>T	p.Ser458Leu	TMEM43-related ARVC shows a sex-influenced malignant phenotype, with earlier and more severe expression in males.
**Milting H, EHJ 2015 [9]**	To evaluate the presence of the missense mutation TMEM43 in a German ARVC family	11 pts with a TMEM43 mutation	c.1073C>T	p.Ser458Leu	TMEM43 screening is recommended in ARVC patients; the mutation may cause nuclear stiffness and cell death.
**Hodgkinson KA, Circ Arrhythm Electrophysiol 2016 [10]**	To assess the long-term outcomes after ICD implantation in ARVC patients with the TMEM43 mutation	148 affected pts (genetic mutation, *n* = 146; VF or SCD, *n* = 2) who underwent ICD implantation	c.1073C>T	p.Ser458Leu	ICDs are recommended in young males with TMEM43; in females, delayed implantation may be appropriate.
**AbdelWahab A, J Cardiovasc Electrophysiol 2018 [11]**	To compare the VT ablation outcomes in ARVC patients with vs. without a TMEM43 mutation	5 pts with a TMEM43 mutation	c.1073C>T	p.Ser458Leu	TMEM43-related ARVC shows a more severe morphology and higher arrhythmic burden during ablation.
**Dominguez F, Heart Rhythm 2020 [12]**	To evaluate the phenotype, clinical course, and impact of exercise in pts with a TMEM43 mutation in non-Newfoundland-related families	62 affected pts (genetic carriers, *n* = 30; obligate carriers, *n* = 14; SCD, *n* = 18)	c.1073C>T	p.Ser458Leu	TMEM43 causes severe ARVC, particularly in males; vigorous exercise may worsen arrhythmias, especially in females.
**Paulin FL, Heart Rhythm 2020 [13]**	To evaluate the impact of high-level physical activity in TMEM43-p.S358L ARVC patients after an ICD for primary prevention	80 pts with a TMEM43 mutation	c.1073C>T	p.Ser458Leu	High-intensity exercise increases the risk of malignant VAs and earlier ICD shock in TMEM43 ARVC patients.
**Ma C, Cardiovasc Ultrasound 2021 [14]**	To assess the longitudinal myocardial strain in a family with TMEM43 mutations	8 pts with a TMEM43 mutation	NA	NA	TMEM43 mutation carriers showed impaired LV layer-specific longitudinal strain vs. that in healthy controls.
**Matos J, Radiol Cardiothorac Imaging 2023 [15]**	To describe the CMR findings in ARVC patients with TMEM43 pathogenic or VUS variants using the 2020 Padua Criteria	14 ARVC pts with a pathogenic variant (*n* = 8) and a VUS (*n* = 7) of TMEM43	c.1073C>T and other VUS	p.Ser458Leu and other	TMEM43-related ARVC shows progressive LV dysfunction and subepicardial fibrosis, requiring longitudinal clinical and imaging follow-up.
**Bueno Marinas M, Int J Mol Sci 2024 [4]**	To assess rare genetic variants in non-desmosomal genes in pts with arrhythmogenic cardiomyopathy	6 pts with a TMEM43 mutation	c.1073C>T; c.349A>G	p.Ser458Leu; p.Arg117Gly	TMEM43 showed 3.8-fold enrichment; one-third of ALVC patients had rare non-desmosomal variants.
**Cabrera-Borrego E, Circ Arrhythm Electrophysiol 2024 [16]**	To evaluate the genotype–substrate correlation in LV-involved inherited cardiomyopathies	6 pts with a TMEM43 mutation	c.1073C>T	p.Ser458Leu	In TMEM43 mutation carriers, the arrhythmic substrate is localized in the RV outflow tract, with biventricular involvement, an LBBB pattern, and LV fibrosis. Nuclear gene mutations are associated with higher arrhythmic and HF risk.

HGVSc: Human Genome Variation Society—coding DNA; HGVSp: Human Genome Variation Society—protein; ICD: implantable cardioverter–defibrillator; SCD: sudden cardiac death; VT: ventricular tachycardia; ARVC: arrhythmogenic right ventricular cardiomyopathy; VAs: ventricular arrhythmias; LV: left ventricular; CMR: cardiac magnetic resonance; VUS: Variant of Uncertain Significance; ALVC: arrhythmic left ventricular cardiomyopathy; RV: right ventricular; LBBB: left bundle branch block; HF: heart failure; NA: not applicable.

## Data Availability

All of the data supporting the findings of this review are contained within the article.

## Abbreviations

The following is a list of the abbreviations used in the text:

ACM	Arrhythmogenic cardiomyopathy
VA	Ventricular arrhythmia
SCD	Sudden cardiac death
TMEM43	Transmembrane protein 43
ECG	Electrocardiogram
EPS	Electrophysiologic study
CMR	Cardiac magnetic resonance
HF	Heart failure
HT	Heart transplant
ICD	Implantable cardioverter–defibrillator
PRWS	Poor R wave progression
PVC	Premature ventricular contraction
SAEG	Signal-averaged ECG
LV	Left ventricular
LVEF	Left ventricular ejection fraction
RV	Right ventricular
LGE	Late gadolinium enhancement
JUP	Plakoglobin
DSP	Desmoplakin
PKP2	Plakofillin
DSG2	Desmoglein 2
DSC2	Desmocollin 2
DES	Desmin
PLN	Phospholamban
FLNC	Filamin C
CDH2	Cadherin 2
TJP1	Tight Junction Protein 1
LBBB	Left bundle branch block
RBBB	Right bundle branch block
VT	Ventricular tachycardia
